# Isolation and characterization of eight microsatellite loci from *Galeocerdo cuvier* (tiger shark) and cross-amplification in *Carcharhinus leucas, Carcharhinus brevipinna*, *Carcharhinus plumbeus* and *Sphyrna lewini*

**DOI:** 10.7717/peerj.2041

**Published:** 2016-05-17

**Authors:** Agathe Pirog, Sébastien Jaquemet, Antonin Blaison, Marc Soria, Hélène Magalon

**Affiliations:** 1 UMR ENTROPIE, Université de la Reunion, Saint Denis, La Réunion; 2 UMR MARBEC, IRD Réunion, Saint Denis, La Réunion; 3 Laboratory of Excellence CORAIL, France

**Keywords:** Carcharhiniform, Microsatellites, Control region, Population Genetics

## Abstract

The tiger shark *Galeocerdo cuvier* (Carcharhinidae) is a large elasmobranch suspected to have, as other apex predators, a keystone function in marine ecosystems and is currently considered Near Threatened (Red list IUCN). Knowledge on its ecology, which is crucial to design proper conservation and management plans, is very scarce. Here we describe the isolation of eight polymorphic microsatellite loci using 454 GS-FLX Titanium pyrosequencing of enriched DNA libraries. Their characteristics were tested on a population of tiger shark (*n* = 101) from Reunion Island (South-Western Indian Ocean). All loci were polymorphic with a number of alleles ranging from two to eight. No null alleles were detected and no linkage disequilibrium was detected after Bonferroni correction. Observed and expected heterozygosities ranged from 0.03 to 0.76 and from 0.03 to 0.77, respectively. No locus deviated from Hardy-Weinberg equilibrium and the global *F*_*IS*_ of the population was of 0.04^*NS*^. Some of the eight loci developed here successfully cross-amplified in the bull shark *Carcharhinus leucas* (one locus), the spinner shark *Carcharhinus brevipinna* (four loci), the sandbar shark *Carcharhinus plumbeus* (five loci) and the scalloped hammerhead shark *Sphyrna lewini* (two loci). We also designed primers to amplify and sequence a mitochondrial marker, the control region. We sequenced 862 bp and found a low genetic diversity, with four polymorphic sites, a haplotype diversity of 0.15 and a nucleotide diversity of 2 × 10^−4^.

## Introduction

The tiger shark *Galeocerdo cuvier* is a large carcharhinid (up to 5.5 m in total length for the largest females), which lives in warm temperate, tropical and subtropical waters (Compagno, 1984; Randall, 1992). This species is opportunistic and feeds on a very large range of preys according to their availability in the environment. It is considered as a generalist forager (Lowe et al., 1996; Simpfendorfer, Goodreid & McAuley, 2001). Different studies estimated the age of sexual maturity around seven to ten years for both sexes (Branstetter, Musick & Colvocoresses, 1987; Natanson et al., 1999; Kneebone et al., 2008), and a lifespan between 27 and 29 years for males and females, respectively (Kneebone et al., 2008). The species favours coastal habitats (Heithaus et al., 2006; Papastamatiou et al., 2013) and can be highly reef associated (Meyer, Papastamatiou & Holland, 2010) even if transoceanic movements are regularly observed (Rooney et al., 2006; Heithaus et al., 2007; Lea et al., 2015). Tiger sharks occupy defined but very large home ranges (Holland et al., 1999), which renders difficult the implementation of adapted conservation measures. *Galeocerdo cuvier* is classified as Near Threatened by the International Union for Conservation of Nature (IUCN) Red List of Endangered Species (Simpfendorfer, 2009). Although it is one of the largest marine predators, little is known about the ecology of this species, which has mostly been studied using *in situ* and direct observations. To our knowledge, only one study has focused on population genetics of *G. cuvier*, in Hawaii (Bernard, Feldheim & Shivji, 2015), and characterized nine microsatellite loci for this species. Another study (Chen et al., 2014) mapped the entire mitogenome for this species but did not test for mitochondrial markers that would be useful for population genetics studies.

Here, we developed a supplementary set of eight microsatellite loci for the tiger shark, polymorphic at the population scale. Until now, 12 microsatellite loci were available for the tiger shark, including the nine developed in Bernard, Feldheim & Shivji (2015) and the three loci characterized from *Carcharhinus leucas* that are polymorphic in *G. cuvier* (Pirog et al., 2015). With these eight new loci, 20 microsatellite loci will now be available to study this species, which will be very useful when studying the genetic structure of tiger shark populations worldwide. We described their characteristics by genotyping 101 *G. cuvier* individuals caught at Reunion Island, South-Western Indian Ocean, and tested cross-amplification in four other carcharhiniform species. Furthermore, we designed primers to amplify and sequence the mitochondrial control region (also called D-loop).

## Material and Methods

The samples were collected on tiger sharks caught during the scientific program CHARC (French acronym for “Knowledge on the ecology and the habitat of two coastal shark species”) off the west coast of Reunion Island (21°06′S, 55°36′E), South Western Indian Ocean, 700 km east from Madagascar between October 2011 and May 2013 (Blaison et al., 2015). The program CHARC was approved by Reunion Island Ethic Committee, the local representative of the French national ethic committee. For sharks tagged during an acoustic study (Blaison et al., 2015), a piece of fin tissue was biopsied on living animals. Pieces of muscle were also collected from professional fishermen by-catches. A total of 101 adults (46 males and 55 females) were sampled.

Total genomic DNA was extracted using Qiagen DNeasy Blood & Tissue kit (Qiagen, Hilden, Germany) from small pieces of tissues (fin or muscle). The microsatellite library was developed using 11 individuals (six females and five males). Biggest tagged individuals (320–390 cm total length) were chosen to decrease the probability of sampling related individuals and thus, to increase genetic variability. Indeed, choosing both small and big individuals increases the probability to use related individuals (parents and their offspring) to construct the library. Total genomic DNA was sent to GenoScreen, Lille, France (www.genoscreen.fr). One µg was used for the development of the microsatellites library through 454 GS-FLX Titanium pyrosequencing of enriched DNA libraries as described in Malausa et al. (2011). Briefly, total DNA was mechanically fragmented and enriched for AG, AC, AAC, AAG, AGG, ACG, ACAT and ATCT repeat motifs. Enriched fragments were subsequently amplified. PCR products were purified, quantified and GsFLX libraries were then carried out following manufacturer’s protocols and sequenced on a GsFLX PTP. Sequences of the microsatellite-enriched library were analysed using the software QDD (Meglecz et al., 2010) and primer pairs were selected depending on the motif (di-, tri-, tetra-, hexanucleotide), the number of repeats (≥5) and the product size (≥100 bp) and tested on agarose gel for amplification. Then, depending on the putative allele number, the polymorphism was verified by genotyping 11 *G. cuvier* individuals on an ABI 3730 XL sequencer (Applied Biosystems, Foster City, CA, USA).

The developed set of microsatellite loci was then used to genotype our sampling of 101 *G. cuvier* individuals. Each amplification reaction contained 10 µL of PCR product. Microsatellite loci developed in this study were directly fluorochrome labelled (using 6-FAM, PET, VIC or NED), and the reaction mixture contained 5 µL of MasterMix Applied 2× (Applied Biosystems, Foster city, CA, USA), 1.5 µL of demineralized water, 0.5 µL of each primer (10 µM) and 2.5 µL of genomic DNA (10 ng/µL). The thermocycling program was: an initial denaturing step at 94 °C for 5 min, 7 cycles of 94 °C for 30 s, 62 °C (−1 °C at each cycle) for 30 s, 72 °C for 30 s, 35 cycles of 94 °C for 30 s, 55 °C for 30 s, 72 °C for 30 s, followed by a final extension step at 72 °C for 5 min. Allelic sizes were determined using Genemapper v 4.0 (Applied Biosystems, Foster city, CA).

The transferability of these loci was checked on the bull shark *Carcharhinus leucas* (*n* = 41), the spinner shark *Carcharhinus brevipinna* (*n* = 2), the sandbar shark *Carcharhinus plumbeus* (*n* = 3) and the scalloped hammerhead shark *Sphyrna lewini* (*n* = 4). These samples have been collected from professional fishermen by-catches caught at Reunion Island. Extractions and genotyping were conducted following the same protocol as above.

From the *G. cuvier* mitochondrion sequence available in GenBank (KF111728.1), we designed primers to amplify the control region (D-loop) using PRIMER3 v 4.0.0 (Rozen & Skaletsky, 2000): the forward primer Gc-CR-F (5’-CCC AAA GCC AAG ATT CTG CC-3’) and the reverse primer Gc-CR-R (5’-CGA GAC CAA CCA TGT ATA TTA AGG G-3’). These primers were used for both amplification and direct sequencing. PCR reactions were performed in a total volume of 25 µL containing 12.5 µL of Applied MasterMix 2x (Applied Biosystems, Foster city, CA), 7.5 µL of demineralized water, 1 µL of each primer (10 µM) and 3 µL of genomic DNA (10 ng/µL). The thermocycling program contained an initial denaturing step at 94 °C for 5 min, 35 cycles of (94 °C for 30 s, 56 °C for 30 s, 72 °C for 1 min 30 s), and a final extension step at 72 °C for 5 min. The sequencing of the mitochondrial DNA was realised by GenoScreen, Lille, France (www.genoscreen.fr).

Concerning microsatellite loci, presence and frequencies of null alleles, which may be responsible for an excess of homozygotes, were assessed using MicroChecker v 2.2.3 (Van Oosterhout et al., 2004). Tests of linkage disequilibrium were performed using Arlequin v 3.5.1.2 (Excoffier & Lischer, 2010). Diversity indices such as the number of alleles per locus *N*_*a*_, the observed and expected heterozygosities (*H*_*O*_ and *H*_*E*_), the inbreeding coefficient *F*_*IS*_ (Cockerham & Weir, 1984) were assessed, and Hardy-Weinberg equilibrium was tested using FSTAT v 2.9.3.2 (Goudet, 1995).

Mitochondrial sequences were edited and aligned using Geneious v 6.1.7 created by Biomatters (available from http://www.geneious.com/). Haplotype and nucleotide diversities were calculated using DnaSP v 5.10.1 (Librado & Rozas, 2009).

## Results

Sequencing of the microsatellite-enriched library yielded 20,303 reads. A total of 6,982 sequences (34%) containing microsatellite motif were identified. After QDD analysis, 103 primer pairs were recognized. Among these, 95 primer pairs were selected depending on our criteria: 36 (37.9% ) successfully amplified. Then, depending on the putative allele number, the polymorphism of 32 primer pairs was verified by genotyping 11 *G. cuvier* individuals on an ABI 3730 XL sequencer. A total of eight microsatellite loci (GenBank accession numbers: KP300805, KP300806, KP300807, KP300808, KP300809, KP300810, KP300811, KP300812) were finally selected and characterized for *G. cuvier* (Table 1).

**Table 1:** Characterization of the eight microsatellite loci developed for *Galeocerdo cuvier* and their primer sequences (F: forward; R: reverse). No locus deviated from Hardy-Weinberg equilibrium. Annealing temperature *Ta* = 55 °C.

Locus name	Repeat motif	Forward primer (5′–3′)	Reverse primer (5′–3′)	Allele size (bp)	*N*_*a*_	*H*_*O*_	*H*_*E*_	*F*_*IS*_
Gc01	(GA)_6_	AGGTGTGGTGGCTCTCCTC	GGACGCAAAATCCAACAGAG	143–147	2	0.03	0.03	− 0.01
Gc02	(AG)_7_	GAGAGGGAGAAGCAAGTCAACATA	GTTTCTCTTCTTGTCCTCTTCCA	93–105	4	0.15	0.15	0.01
Gc03	(TC)_8_	TTGATTTCTACCTGGTCGGC	TCAGAGCAAAGAGCTCCAGA	121–129	3	0.56	0.58	0.04
Gc04	(TC)_9_	CCCCAGGGAAATAATCTAAGG	CAGGGGGACGACTAGTCAAG	195–199	2	0.36	0.38	0.07
Gc05	(CT)_11_	CTGGGTGGCAGCAAATTAGA	TGAGCCTTCTCACCCAGAGT	117–123	2	0.45	0.50	0.10
Gc06	(AC)_11_	CATGACGTTTCGCCACAATA	TTTCCTCCCACAGTCCAAAG	116–122	3	0.13	0.14	0.07
Gc07	(CA)_14_	ATTGCAATCTGTGCCATCAA	TTTGTGAGAGTGTCTGTATGTTTG	114–130	8	0.76	0.77	0.02
Gc08	(AGTG)_6_	GTGCAGGGAGGAATGTGAGT	TTGTCAAGAGTCCACGTGTCTT	207–219	3	0.49	0.49	0.02

**Notes.**

Diversity indices are issued from FSTAT v2.9.3.2;

bp Base pairs *N*_*a*_ Number of alleles per locus *H*_*O*_ Observed heterozygosity *H*_*E*_ Expected heterozygosity *F*_*IS*_ Inbreeding coefficient

The eight loci developed in *G. cuvier* were then used to genotype 101 tiger sharks caught at Reunion Island between 2011 and 2013. All loci were polymorphic with a number of alleles ranging from two to eight. No null alleles were detected and no linkage disequilibrium was detected after Bonferroni nor FDR corrections. Observed and expected heterozygosities ranged from 0.03 to 0.76 and from 0.03 to 0.77, respectively (Table 1). No locus was found to deviate from Hardy-Weinberg equilibrium and the global *F*_*IS*_ of the population was of 0.04^*NS*^, following Hardy-Weinberg proportions.

**Table 2:** Cross-amplification for eight microsatellite loci designed for *Galeocerdo cuvier* across four Carcharhiniformes: *Carcharhinus leucas* (*n* = 41), *Carcharhinus brevipinna* (*n* = 2), *Carcharhinus plumbeus* (*n* = 3) and *Sphryna lewini* (*n* = 4).

Locus name	*C. leucas*	*C. brevipinna*	*C. plumbeus*	*S. lewini*
	(*n* = 41)	(*n* = 2)	(*n* = 3)	(*n* = 4)
Gc01	+P 136-142 (3)	+139(1)	+135(1)	+P 152–156 (3)
	**41/41**	**2/2**	**3/3**	**4/4**
Gc02	–	–	+P 99-101 (2)	–
			**2/3**	
Gc03	–	–	–	–
Gc04	–	+198(1)	+P 194-200 (2)	–
		**2/2**	**2/3**	
Gc05	–	+159(1)	+105(1)	–
		**2/2**	**2/3**	
Gc06	–	–	+P 112-116 (3)	–
			**3/3**	
Gc07	–	–	–	+P 173-185 (4)
				**4/4**
Gc08	–	+222(1)	–	–
		**2/2**		

**Notes.**

+ Amplified +P Polymorphic − No amplification

Size ranges in base pairs and number of alleles (in parentheses) are also indicated. Numbers of amplifications observed are indicated in bold.

Among these eight loci, one (12.5%) successfully cross-amplified in *C. leucas*, four (50%) in *C. brevipinna*, five (62.5%) in *C. plumbeus,* and two (25%) in *S. lewini* (Table 2). The number of alleles ranged from one to four depending on the species (Table 2).

The 101 *G. cuvier* adults were sequenced, and sequences were edited and aligned over 862 bp. All sequences were of high quality and easily readable without ambiguities. We observed four polymorphic sites (over 862 bp), distributed among only five haplotypes, which were deposited in GenBank (accession numbers: KP317128, KP317129, KP317130, KP317131, KP317132, ). The haplotype diversity (*h*) was 0.15 ± 0.048 (sd) and the nucleotide diversity (*π*) 2 × 10^−4^ ± 7 × 10^−5^ (sd). Among these haplotypes, one was over-represented (*n* = 93; 92% of the individuals sequenced).

## Discussion

The development of these loci (nuclear and mitochondrial), added to those previously described in Bernard, Feldheim & Shivji (2015) and in Pirog et al. (2015) (three loci characterized from *C. leucas* polymorphic for *G. cuvier*), will be very useful in studying tiger shark ecology, which remains poorly documented, especially in assessing population structure and patterns of migration, effective population size and some aspects of their reproductive behaviour. Furthermore, the mitochondrial control region highlighted low genetic diversity in the tiger shark and should be useful to study the evolution of tiger shark populations.

These loci may also be used when studying other carcharhiniform species. Indeed, most shark species being heavily exploited by both artisanal and industrial fisheries, including in the Western Indian Ocean (Campana & Ferretti, 2016), it is relevant and useful to possess genetic tools to conduct population genetics analyses.

## Supplemental Information

10.7717/peerj.2041/supp-1 Data S1 Dataset Microsatellite data and sequences of the control region for individuals studied. (−) indicates no data

## Additional Information and Declarations

### DNA Deposition

The following information was supplied regarding the deposition of DNA sequences: For microsatellite sequences, GenBank accession numbers: KP300805, KP300806, KP300807, KP300808, KP300809, KP300810, KP300811, KP300812. For mitochondrial sequences, Genbank accession numbers: KP317128, KP317129, KP317130, KP317131, KP317132.

### Data Availability

The following information was supplied regarding data availability: The raw data has been supplied as Data S1.
